# Renewing the Sydney undergraduate curriculum

**DOI:** 10.1007/s10734-022-00982-x

**Published:** 2022-12-15

**Authors:** Philippa Pattison, Adam Bridgeman, Ainslie Bulmer, Peter McCallum, Richard Miles

**Affiliations:** Office of the Deputy Vice-Chancellor (Education), University of Sydney, Camperdown, Australia

**Keywords:** Undergraduate curriculum, Graduate attributes, Graduate qualities, Institutional curriculum change

## Abstract

A number of commentators have recently called for a re-examination of the purpose and value of undergraduate education, arguing that change is required if universities are to deliver the value in educational outcomes that students and communities now require for a changing and challenging world (for example, Aoun, 2017; Bok, 2020; Davidson, 2017; Fischman & Gardner, 2022). Indeed, some have argued that such change is necessary to stem an emerging crisis in universities’ ‘social license to operate’ (Bok, 2020). In this paper, we review the case for undergraduate curriculum change and present a case study of one Australian university’s engagement with this challenge, describing the reasons for change, the desired outcomes, and some early impacts on students’ study patterns. The change took place at the University of Sydney over the period from 2014 to 2021 with a new undergraduate curriculum introduced for commencing students from 2018. Intended to prepare students for a changing world, the new curriculum sought a balance between graduates’ expertise in a primary field of study and a set of broader capabilities that would support their capacity for future learning and for creative and effective engagement in life and career, including an understanding of broader intellectual landscapes; the skills for collaboration, invention, and influence; and the integration of knowledge with professional and personal ethics and values. The aspiration to develop such capabilities is shared with many universities around the world, and we describe here how the available evidence base was used to guide whole-of-University curriculum redesign in this case. We also identify areas where further research would be of value.

## Introduction

For many years, undergraduate education has been recognised as offering social and economic value to society and to graduates, including an intergenerational educational benefit for graduates’ children (e.g. Mayhew et al, 2016; US Bureau of Labor Statistics, 2022). Moreover, these benefits have been more widely shared in recent years as rates of undergraduate participation have risen in many countries around the world (e.g. OECD, 2021). However, some commentators have questioned whether the benefits to society and to graduates could be greater, and have urged re-consideration of the educational purpose and approach to undergraduate education (e.g. Aoun, 2017; Bok, 2020; Davidson, 2017; Fischman & Gardner, 2022).

In this paper, we set out the arguments for reviewing the purpose of undergraduate education and present a case study of one Australian university’s engagement with the challenge it presents for curriculum and educational approach. We describe how the higher education literature provided some helpful guidance on educational approaches and describe some initial impacts on students’ study patterns of the changes adopted.

### Why reconsider the purpose of an undergraduate education?

The first reason offered for reconsideration of the purpose of an undergraduate education is that evolving changes in the demands of work and career arising from globalisation and digital transformation now call for a subtly different constellation of graduate capabilities. While graduates will still be expected to have contemporary expertise in their primary field of study, they are also likely to face increasingly complex societal challenges and systemic workplace change. They will likely experience more complex career trajectories as a result and need to update and broaden their expertise on a more regular basis throughout their working lives. These challenges, it is argued, will require capabilities such as agility, adaptability, collaboration, agency, and creativity if graduates are to respond effectively to ongoing change and uncertainty. In Australia, the dynamic nature of future careers was emphasised by the report of the Committee for Economic Development of Australia of the country’s future workforce needs (CEDA, 2015); their predictions mirrored those in other parts of the world (e.g. Frey & Osborne, 2017). A study of national capacity for innovation and entrepreneurship by the Australian Council of Learned Academies identified a greater need for ‘broad relational and problem solving skills applicable across all disciplines’ and suggested that universities should ensure broader study profiles for all students, more internships and practica, and projects that span faculties (Cunningham et al., 2016, p. 11). In the USA, Joseph Aoun (2017) argued for the increasing importance for graduates of critical thinking, systems thinking, entrepreneurship, and cultural agility as well as literacies in technology, data, ethics and collaboration, and highlighted the role of experiential learning in developing these capabilities. Likewise, Davidson (2017) has called for a more active, engaged, and problem-focused educational model that develops a strong capacity to learn throughout life. Importantly, and as Bok (2020) has also argued, the likely dynamic and complex nature of future work and careers highlights the potential value to graduates of personal and interpersonal capabilities such as agency, collaboration, and cross-cultural engagement.

A second reason to re-assess the purpose of undergraduate education is a disturbing set of findings from the USA that, despite the greater challenges facing graduates in the workplace, learning gains during the undergraduate years in vital capabilities such as critical thinking and writing appeared to be in decline compared to prior decades (Arum & Roksa, 2011). These findings echoed earlier quality concerns raised by Bok (2006). Furthermore, low skills gained during an undergraduate degree were demonstrated to translate into poorer employment outcomes (Arum & Roksa, 2014). Together with earlier evidence that the trajectory of learning established during an undergraduate degree continues on a similar course in the years immediately following graduation (Pascarella & Terenzini, 2005), these findings signal the importance of purpose and challenge during the undergraduate years. More recently, Fischman and Gardner (2022) have also called for the reframing and reconfirmation of the purpose of an undergraduate education. In a study involving interviews with more than 2000 students across 10 diverse US campuses, they found that 45% of students had a transactional view of their undergraduate experience, seeing it simply as a milestone towards achieving their ambitions for graduate school or work rather than as an opportunity for deep learning, exploration, or personal transformation. Fischman and Gardner concluded that universities need not only to re-assess their undergraduate educational mission but also to engage more explicitly and regularly with their students about their mission if it is to be fully achieved.

A third argument for review is the emerging understanding of the important neurocognitive and psychosocial developments that occur in the period of late adolescence and early adulthood that overlaps substantially with the typical undergraduate years, especially in research-intensive universities who typically admit a high proportion of undergraduate students directly from secondary school (Bok, 2020; Thompson, 2014). As Thompson (2014) argued, major changes occurring during this period are in brain regions that support higher-order capabilities such as the co-ordination of thought and behaviour, self-awareness, perspective taking, identity formation, and self-regulation. Thompson (2014) stressed the developing integration of cognition and affect during this period and highlighted the role of ‘high-impact’ educational practices (Kuh, 2008), such as projects, service learning, and intercultural experiences, in fostering the development of these broader capabilities during this period. The increasing demand for personal and interpersonal capabilities associated with more dynamic workplaces and careers also reinforces the importance of attending to these higher-order capabilities during the undergraduate years.

A fourth reason for review of educational purpose is suggested by data on graduate outcomes. Although, in 2017, national Australian survey data indicated that 81% of current Australian university students were satisfied with their skill development (QILT, 2017a) and 84% of employers were satisfied with recently hired graduates (QILT, 2017b), some skill areas were seen to be weaker than others and there remained scope for improvement. The areas employers saw as most in need of development were skills for collaboration and for performing and innovating in the workplace; students were themselves least satisfied in relation to skills for communication, teamwork, complex problem-solving, and work-related knowledge and skills. Moreover, as the annual national report on graduate outcomes regularly demonstrates, full-time employment outcomes for bachelor graduates in Australia are modest in the short term (69% four months post-graduation in 2021) though substantially better in the medium term (89% three years post-graduation in 2021; QILT, 2021a, 2021b). In the USA, the recent Association of American Colleges and Universities (AAC&U) Employer Report (Finney, 2021) also rated the ability to work in teams as the graduate capability most valued by employers, and indicated some still sizable gaps in the importance ascribed by employers to certain graduate capabilities and their perceptions of graduate preparedness. The most notable of these gaps were for critical thinking, the application of knowledge and skills in real world settings, analysis and interpretation of data, complex problem-solving skills, and the capacity to work effectively in teams.

Taken together, these arguments recognise the ongoing importance of developing expertise in a chosen field of study, but point to the value of attending carefully, first, to the quality of educational outcomes and, second, to the possibility of more systematic development of a broader set of capabilities that, in combination with core expertise in a field, serve to prepare students more effectively for their lives and careers ahead while also meeting the needs of society. As suggested in the case study below, this is arguably in part a question of curriculum design and in part a question of educational approach.

It is important to recognise in this discussion, of course, that different considerations may be relevant across different higher education systems, institutions, and degrees. One particularly salient degree characteristic in the Australian context is the extent to which it serves as a recognised pathway to a professional career or seeks to provide a broader education more in the style of the liberal arts. As Davis (2017) has argued, most Australian universities offer a similar and comprehensive mix of Bachelor programs including a suite of popular vocationally oriented professional and specialised degrees (e.g. engineering, law, teaching) as well as a collection of broader degrees (e.g. arts, science, business). The arguments summarised above may lead to different analyses for these different types of degree. For example, professional degrees tend to have relatively fixed programs of study that prepare students for initial entry into the profession, but may have little room for broadening activities that prepare students for later systemic change. Broader degrees, on the other hand, may afford diverse opportunities for students to enjoy deep and experiential learning, but not all students will necessarily choose to take them up.

### Undergraduate curriculum review

A number of universities have undertaken whole-of-University curriculum reviews in the last 15 or so years and these processes serve as useful context for the process of review to be described in this paper. In Australia, the University of Melbourne radically transformed its educational offerings from 2008 to offer a small number of broadly based undergraduate degrees promoting both depth and breadth of learning in the undergraduate years and a suite of professional and specialised postgraduate degrees (Emison, 2013; James & McPhee, 2012). The University of Western Australia followed with a similar model several years later (see Woelert et al., 2022, for a comparison). In the USA, recent reform has focussed primarily on the General Education component of undergraduate degrees (e.g. University of Virginia, Harvard University) with attention to breadth of knowledge and modes of inquiry and experience in addressing real-world problems from multiple disciplinary perspectives. In the UK, the University of Aberdeen introduced an Enhanced Study Program in its undergraduate degrees, including a suite of ‘Sixth Century Courses’ to introduce students to multiple disciplinary perspectives on contemporary issues. There are likely many other examples as well but what these initiatives appear to share is an interest in re-developing those aspects of the curriculum that serve to provide broader experience and context for graduates’ disciplinary expertise as well as capabilities for integrating perspectives beyond the primary field of study.

In the remainder of the paper, we provide some background on the University of Sydney and its context, and present an account of the University’s discussion on educational purpose. We then describe the agreed strategies for curriculum re-design and educational approach and point to the scholarly literature that guided their choice. In the final sections of the paper, we present some early indicators of change in study patterns and identify where further research would be of value.

### Context: the University of Sydney

The University of Sydney is a public research-intensive university founded as Australia’s first in 1850. In 2014 when re-consideration of the purpose of an undergraduate education was first discussed, the University had an undergraduate cohort of around 33,000 students enrolled in over 120 undergraduate degrees offered across 16 faculties. The undergraduate students made up 64% of the total student body in 2014 and just over one-third of the undergraduate students were international. By 2021, the undergraduate cohort had grown to around 41,000 students, largely through growth in international student numbers. Undergraduate domestic student enrolments at the University are relatively stable from year to year, largely because of a fixed annual envelope per institution for the total Commonwealth government contribution to domestic student fees. The University of Sydney tends to attract high-achieving undergraduate students, most of whom are school leavers, selecting students primarily on the basis of an Australian Tertiary Admission Rank but also taking account of personal and educational disadvantage. This means that, like other research intensive universities in Australia, approximately 75% of students are 19 years of age or less on commencement. Approximately 60% of commencing undergraduate students enrol in broadly based degrees in arts, business, and science each year; a further 8% enrol in a broadly based degree and a professional pathway degree; and the balance of just over 30% enrol in a degree offering a pathway to a professional career. National higher education data, including the suite of surveys and data collectively termed Quality Indicators of Learning and Teaching (QILT), show that the University of Sydney has high rates of retention and positive employment and further study outcomes for Bachelor graduates, but is rated much less positively for the student experience. The University had not previously undertaken a wide-ranging and whole-of-institution review of the purpose of undergraduate education. The four reasons canvassed above as well as the relatively low ratings of students’ undergraduate experience were broadly accepted as a compelling basis for review.

## Reviewing undergraduate education at the University of Sydney

Discussion of educational purpose for undergraduate degrees at the University commenced with the senior leaders of the University and then through a series of discussions across the University over the course of a year, including through consultation and development of the University’s 2016–2020 strategic plan. A series of discussion papers were developed initially to seed discussion, provide relevant data and research, and then capture the emerging proposals for change. Staff and students were also surveyed, and invited to numerous focus groups and faculty meetings to offer views on a range of issues. Alumni and business and community leaders were also consulted. In response to the survey, students indicated a desire for more opportunities to develop as global citizens, engage with ‘real world’ settings, discuss ethical questions in their study domains, study subjects from other faculties, and engage in interdisciplinary and inter-professional units of study. Many identified ‘limited subjects and content’ as a principal barrier to achieving their educational goals. For their part, staff supported strategies to better contextualise disciplinary understanding, create more systematic opportunities for students to engage with real-world settings and global challenges, and simplify the University’s degree profile to enable broader study.

The discussions settled on several propositions related to educational purpose that were captured in the University’s 2016–2020 strategic plan (University of Sydney, 2016). A primary commitment was to agree on a shared educational purpose by adopting a suite of *graduate qualities* for Bachelor graduates, namely, a set of capabilities to be developed through a structured sequence of learning activities and to be embedded, in an appropriately contextualised way, in the learning outcomes of each of the University’s Bachelor degrees. It was also agreed to simplify the University’s undergraduate degree structure in order to broaden students’ access to disciplines outside their primary field, expand students’ access to experiential and broadening learning opportunities, and adopt a broader, shared commitment to interactive and collaborative pedagogies (Laurillard, 2012) .

### Graduate qualities and the purpose of an undergraduate education

A strong and shared aspiration to emerge from the discussion of educational purpose was that graduates should not just be experts in their fields but also have the capabilities to continually extend their expertise and to use it ‘for good’. Detailed discussion identified and sharpened a list of constituent capabilities. The development of the graduate qualities was seen as interwoven with the development of mastery in a chosen field of study, and to be acquired primarily through a disciplinary lens and an emerging understanding of the contexts for disciplinary practice but, importantly also, through broader experiences in other disciplinary or multidisciplinary contexts. As explained below, the proposal for graduate qualities was accompanied by a curriculum framework which sought to elaborate for each quality the kinds of educational experiences that had been demonstrated to develop it. Needless to say, the evidence base for the development of these qualities was neither comprehensive nor unambiguous, but the curriculum framework was nonetheless grounded in the evidence available at the time (e.g. as summarised in Pascarella & Terenzini, 2005).

The chosen graduate qualities are not unique to the University of Sydney and indeed overlap with the various higher level learning outcomes, or graduate attributes, to which many universities aspire. The University of Sydney had possessed an agreed and carefully conceptualised set of graduate attributes for some time (Barrie, 2003) but the introduction of new graduate qualities signalled a more systematic, future-oriented, and designed approach to their development. It did so, first, through an agreement to embed graduate qualities, appropriately contextualised, among the learning outcomes of every degree, second, by the adoption of the curriculum framework, and, third, and a little later, by the development of an assessment plan for each degree, stream, and/or major, setting out the ways in which each developed and assessed the graduate qualities.

The shared nature of higher level learning outcomes has been observed in a number of higher education systems, including in Australia (Oliver, 2011) and the UK (Wong et al., 2021). In the USA, and reflecting a commitment to the ideals of a liberal education, the AAC&U developed through extensive consultation an agreed set of Essential Learning Outcomes (ELOs) for undergraduate degrees covering knowledge of human cultures and the physical and natural world, intellectual and practical skills, personal and social responsibility, and integrative and applied learning (AACU, 2007) . The ELOs are currently under review (AACU, 2022) .

The final suite of graduate qualities identified by the University of Sydney is presented in Table 1. The list includes depth of disciplinary expertise as a core commitment to rigorous preparation in a primary field of study and the core academic capabilities of critical thinking and problem-solving, communication (oral and written), and digital and information literacy. The inclusion of inventiveness reflects the growing importance of creativity, innovation, and entrepreneurship for effective response to change and uncertainty, just as Aoun (2017) also argued. Cultural competence includes the capacity to work effectively with others across cultural boundaries, an understanding of Aboriginal and Torres Strait Islander cultures and knowledge systems and a mature appreciation of contemporary issues for Aboriginal and Torres Strait Islander peoples. It is recognised as vital to the capacity to work effectively in diverse teams and settings. Interdisciplinary effectiveness is the capacity to work across disciplinary boundaries, and reflects the expectation that many significant challenges to confront graduates will require them to work productively with others possessing quite different forms of expertise. An integrated personal, ethical, and professional identity (Thompson, 2014) captures the capacity to recognise and reflect on personal and cultural value systems and to understand the role of values and ethics in the reasoning of self and others. Its inclusion recognises late adolescence and early adulthood as an important period for identity formation and the role of the University to ensure that students are aware of relevant ethical systems and develop the tools to analyse and respond to differences of perspective, value, or ethics. Finally, influence is conceived as the capability to exert agency when required, and hence to take responsibility for improving circumstances in the community or the workplace and engaging others positively and effectively.

**Table 1:** The University of Sydney Bachelor graduate qualities and their components

Graduate quality	Definition	Components
Depth of disciplinary expertise	The ability to integrate and rigorously apply the knowledge, understanding, and skills of a recognised discipline defined by scholarly activity, as well as familiarity with the evolving practice of the discipline	Understanding of the conceptual space of a recognised discipline; integration and rigorous application of disciplinary knowledge; awareness of the norms, culture and practice of the discipline; capabilities to participate in the evolving practice in the discipline
Critical thinking and problem solving	The questioning of ideas, evidence, and assumptions in order to propose and evaluate hypotheses or alternative arguments before formulating a conclusion or a solution to an identified problem	Definition of problem or issue in context; critical questioning of ideas, evidence and assumptions; creation and evaluation of hypotheses or alternative arguments; formulation of defensible conclusions and best possible solutions
Communication (oral and written)	The clear exchange of meaning in a manner that is appropriate to the audience and context	Clear conveyance of meanings in terms original to the student; adjustment according to audience and context; use of media and modes appropriate to each communication; clarity of structure and organization of ideas
Information and digital literacy	The ability to locate, interpret, evaluate, manage, adapt, integrate, create, and convey information using appropriate resources, tools, and strategies	Location, interpretation, and evaluation of data and information; management of data and information; adaptation, integration, and conveyance of data and information; creation of data and information; effective use of digital resources, tools, and strategies
Inventiveness	The ability to generate novel ideas and solutions	Reimagines and reframes disparate ideas, observations, or resources; creates novel, ideas, solutions, or actions
Cultural competence	The ability to actively, ethically, respectfully, and successfully engage across and between cultures. In the Australian context, this includes and celebrates Aboriginal and Torres Strait Islander cultures, knowledge systems, and a mature understanding of contemporary issues	Awareness of one’s own cultural values and worldview; actively seeking to understand norms and values of other cultures
Interdisciplinary effectiveness	The integration and synthesis of multiple viewpoints and practices, working effectively across disciplinary boundaries	Understanding of multiple viewpoints and practices; working effectively across discipline and professional boundaries; integrating and synthesising different ways of thinking; production of distinctive outcomes
An integrated professional, ethical, and personal identity	Understanding the interaction between one’s personal and professional selves in an ethical context	Articulates a coherent ethical framework; reflects on the self in personal and professional contexts
Influence	Engaging others in a process, idea, or vision	Responsibility for improvement through involvement and leadership; confidence, self-awareness, and a willingness to learn from others; persuasiveness

The definitions of the graduate qualities provided in Table 1 were developed by the University’s Assessment Advisory Group, a group of discipline experts who oversaw the development of rubrics that could be used to assess each of them. Also included in Table 1 are the key components of each quality that were identified by the group to support their development and assessment.

### Developing the graduate qualities

Adopting the graduate qualities entailed embedding them in a suitably contextualised form as learning outcomes in every degree. The relatively fixed character of the professional degrees meant that carefully targeted changes could be made with impact across all students in the degree. For instance, a shared inter-professional learning requirement was agreed and developed across all health degrees. For the broader degrees with their many majors, at least some of the curriculum redesign work needed to be undertaken at the level of the major. A substantial strategic funding envelope was committed to support the necessary curriculum redesign.

In addition, a shared curriculum framework, conceptualised as a set of curriculum components and educational experiences that would support development of the graduate qualities, was proposed on the basis of the higher education literature and agreed across the institution. Although there is not a simple, one-to-one mapping from the components of the curriculum framework to the graduate qualities, each has been demonstrated to have an impact on relevant forms of student learning and development, as we describe below. An important source of evidence at the time of the development of the curriculum framework was Pascarella and Terenzini’s (2005) wide-ranging synthesis of higher education research. This volume has since been further updated by Mayhew et al. (2016) and we refer to both below. Given the large-scale nature of the change being contemplated, there was strong interest in these and other meta-analytic and synthetic studies that sought to summarise the broad impacts of various educational approaches. Bok (2020) has also provided a recent insightful synthesis of evidence for educational approaches to developing many of these broader capabilities.

Although there is some important subject matter relevant to the development of broader capabilities—knowledge of other cultures, team dynamics, ethical frameworks, or coding, for example—many of the experiences that create the opportunity for broader capability development are those that challenge students to reflect on the application of knowledge and, in some cases, on the limits of its applicability, and to be open to and reflect on the perspective of others. The challenge may come from interpersonal engagement in a collaborative or group-based learning context where there is a need to engage, explore, discuss, listen, think, or act in the moment and in dialogue with others. Alternatively, it may come from applying knowledge in authentic, richly contextualised settings, where the complexity of circumstances often creates dilemmas and challenges and hence a need to innovate or balance competing considerations. As a result, some of the components of the curriculum framework described below overlap with the University’s commitment to broader adoption of the interactive and collaborative pedagogies that have been demonstrated to engage students and promote more effective learning (e.g., Mayhew et al, 2016).

The core components of the curriculum framework intended to develop each graduate quality are summarised in Table 2 and discussed in turn below.

**Table 2:** The curriculum framework (University of Sydney, 2016)

Graduate quality	Curriculum framework core components
Depth of disciplinary expertise	• Required major/stream • Structured approach to developing knowledge, skills, and methods of enquiry • Authentic problems and assessment • One or more projects
Critical thinking and problem solving; communication; information and digital literacy, inventiveness	• Structured approach to developing each capability at key points in the curriculum • One or more projects • Interdisciplinary learning experiences that contextualise disciplinary knowledge and skills • Authentic problems and assessment • Collaborative and group-based learning activities and assessments • Short modular Open Learning Environment (OLE) units on foundational concepts and methods in other disciplines
Cultural competence	• Structured opportunity for the development of cultural competence at key points in the curriculum, including for professional practice • Learning activities that take advantage of the cultural diversity within the University community • Collaborative and group-based learning activities and assessments
Interdisciplinary effectiveness	• Interdisciplinary/inter-professional learning experiences in real-world settings • Authentic problems and assessment • OLE units
An integrated professional, ethical, and personal identity	• Structured opportunities for the development of ethical reasoning in every major/stream • Authentic problems and assessment • One or more projects • OLE units
Influence	• OLE units (e.g. teamwork, team leadership, project management, systems thinking) • Team leadership development in group project settings • One or more projects • Interdisciplinary/inter-professional learning experiences in real-world settings

A required major or specialisation is clearly not a novel requirement but is important in developing depth of expertise in a field, including a coherent and integrated body of knowledge in the field and its methods of enquiry. It has been demonstrated to stimulate broad cognitive development as well as subject matter expertise (Mayhew et al, 2016). A structured approach to the development of knowledge, skills, and methods of enquiry provides clarity of purpose for students and teachers and underpins a coherent and effective curriculum. By promoting student learning, it supports the development of deeper forms of expertise in the primary field while also building broader capabilities (Mayhew et al, 2016; Smith & Baik, 2021).

Collaborative and group-based learning activities and assessments involve students as active and collaborative participants in exploration, discussion, and inquiry, including in group projects in which each student has a specific responsibility. These activities support deeper learning as well as the broader capabilities of critical thinking, reflective thinking, epistemological maturity, self-reported leadership skills, and the capacity to influence others and work effectively in groups (Mayhew et al, 2016; Pascarella & Terenzini, 2005). Similar outcomes have also been confirmed longitudinally, with positive impacts including critical thinking, the inclination to inquire and life-long learning, intercultural effectiveness, and socially responsible leadership (Kilgo et al., 2015). Likewise, meaningful learning experiences involving authentic problems and assessments contribute to greater student engagement and learning (Schneider & Preckel, 2017). A substantial authentic project is a potent form of authentic learning experience and is an example of one of the high-impact educational practices (Kuh, 2008) advocated by the AAC&U for the development of their ELOs.

In relation to interdisciplinary and inter-professional learning experiences, Pascarella and Terenzini (2005) point to evidence that exposure to an interdisciplinary curriculum that encourages intellectual connections among ideas across disciplines is associated with growth in reflective thinking and epistemological maturity. They also concluded that growth in students’ cognitive capabilities is aligned with their study choices, and there is therefore a broadening of cognitive capabilities associated with the study of subject matter outside students’ primary fields. Nonetheless, this is an area where further research would be fruitful. As an example of further work of value, Hughes et al. (2016) provide compelling evidence in a health context of the positive impact of inter-professional learning on both inter-professional team practice and patient outcomes.

The Open Learning Environment (OLE) is a suite of short, modular courses to support students’ broader exploration of ideas, concepts, and methods from fields of study other than their own and hence to broaden their cognitive capabilities (Mayhew et al, 2016). Its courses focus on core concepts in a field (e.g. *anxiety and its disorders*, *foundations of quantum computing*, *complexity*, *cryptocurrency*), capabilities (e.g. *coding literacy*, *writing for the digital world*, *aboriginal Sydney*, *disability awareness and inclusivity*), or thematic analysis (e.g. *heath challenges: diabetes*, *global ethics: migration and nation*). The courses are studied in an online or hybrid mode with the support of online resources and activities and often with the support of in-person workshops.

Relevant to each of these curriculum components is the finding that experiences that challenge students and call for integration across disparate ideas, concepts, and fields stimulate change in a wide variety of cognitive capabilities as well as in lifelong learning orientation, intercultural competence, openness to diversity, leadership, citizenship, and moral development (Mayhew et al, 2016).

In addition to the core components, elective enrichment components of the curriculum framework offer opportunities to undertake a second specialisation; engage in research; broaden cultural horizons through mobility, immersive field experiences, and the study of languages or intensive units abroad; engage in co-curricular leadership, innovation, and volunteering projects; and undertake specific OLE units of study covering thematic interdisciplinary challenges, ethical reasoning, digital and data skills, innovation and entrepreneurship, design thinking, advanced communication skills, intercultural understanding, and project management. The impact of these components is also supported by the literature referred to above. In addition, specific experiences have been shown to support specific components: for example, the discussion of ethical dilemmas can stimulate the use of principled moral reasoning and be further enhanced by instruction in philosophical methods of ethical analysis (Pascarella & Terenzini, 2005).

### Simplifying the degree profile and supporting active and experiential learning

A key design challenge for the new undergraduate curriculum was a degree architecture that would preserve the breadth of the University’s offerings but make study choices navigable to students. It was agreed to preserve a mix of professional and broader degrees and to simplify the degree profile by reducing the broader degrees to a smaller number with a common architecture and common rules, and establishing a shared pool of second majors and units accessible from all of them. The final degree profile comprised 25 distinct undergraduate degrees, and 25 options for combined or double degrees.

Students undertaking the broader degrees also have the option of combining their degree with a Bachelor of Advanced Studies, constructed to enable students to broaden and deepen their primary degree through inclusion of a second major and advanced course and project work. As part of the four-year combined degree, students can also select a research-focussed Honours component of up to a full-time year in length in the final year of study (subject to academic performance requirements and completion of a second major).

An enrichment program was established to offer additional challenge to high-achieving students. Attracting approximately 1500 students per year, it offers students several dedicated, challenge-rich units of study, a global mobility scholarship, and a co-curricular enrichment program alongside their chosen degree.

To support the substantial scale up of authentic project opportunities for students, a new Educational Enterprise and Engagement (EEE) team was established. This team developed a common learning and assessment framework for multidisciplinary Industry and Community Project Units and an at-scale model of industry engagement to support a suite of authentic industry and community projects for each cohort of students. Industry and community partner organisations identify challenges and representatives of the organisations serve as subject-matter experts for students who work in multidisciplinary teams compiled by and supported by EEE staff.

The University’s existing Educational Innovation team provided professional learning and educational design support for greater uptake of interactive and collaborative pedagogies and for educational approaches supporting the curriculum framework, including the creation of the OLE. This support was later developed into an ongoing Modular Professional Learning Framework for educators, a suite of short courses offered in a hybrid online and workshop mode and showcasing interactive and collaborative learning activities.

The new curriculum was in place for commencing students in 2018 and the overall process of curriculum renewal and implementation across the undergraduate years spanned the period 2016–2021. Fortunately, much of the design work was completed prior to March 2020 when the COVID-19 pandemic necessitated an urgent move to remote delivery for most degrees.

## Early impacts on students’ study patterns

In this section, we examine student choices in the new curriculum, including their engagement with the more experiential components of the curriculum framework, and their reported experience of interaction in the classroom (to the extent that data are available).

**Fig. 1:**
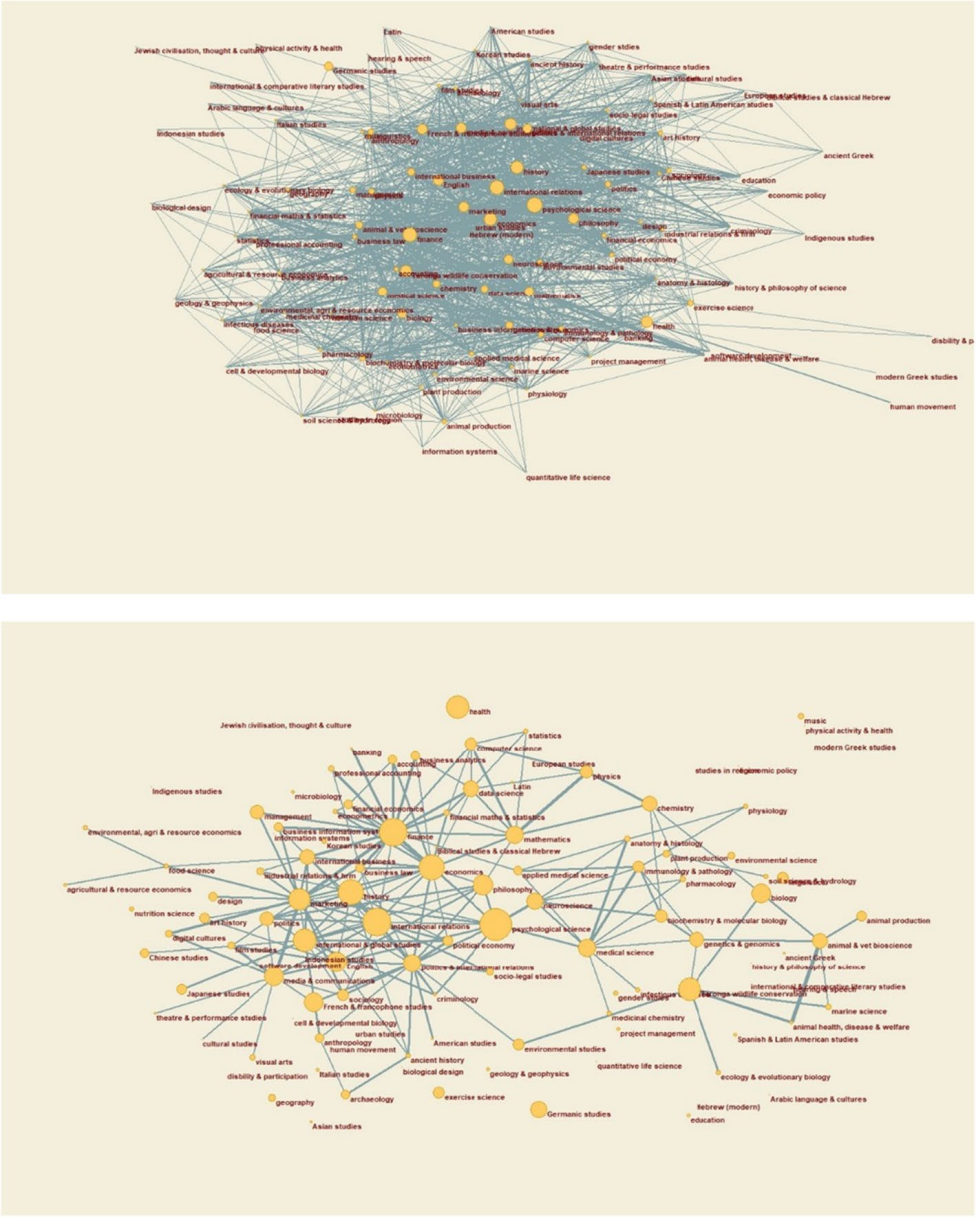
Pairings of majors for students enrolling in a broad three year degree and the Bachelor of Advanced Studies at the University of Sydney, 2018–2021. Two majors are connected if they are jointly chosen by at least 2 students (upper panel) or at least 9 students (lower panel), with the width of the connecting line a logarithmic function of the number who choose both. The size of each node reflects its *betweenness centrality* (Freeman et al., 1991) in the network, that is, the extent to which it sits on shortest paths connecting other pairs of majors

**Fig. 2:**
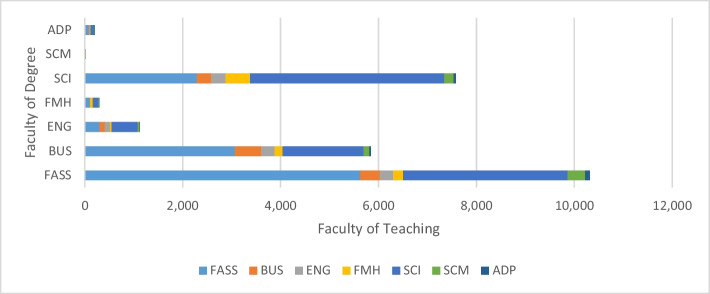
Open Learning Environment enrolments at the University of Sydney in 2021 as a function of faculty of enrolment and faculty of teaching (FASS, arts and social sciences; BUS, business; ENG, engineering; FMH, medicine and health; SCI, science; SCM, music; ADP, architecture, design and planning)

Students enrolled in the new broader degrees appear to have embraced the breadth of choice on offer. For example, the almost 7000 students who had enrolled in a broader degree combined with the Bachelor of Advanced Studies by the end of 2021 had, between them, chosen over 1400 distinct combinations of two majors. Forty-three per cent of these students were enrolled in majors offered by different faculties. Pairings of majors are displayed in Fig. 1 in the form of a network. The upper and lower panels of Fig. 2 reflect 90% and 60% of enrolments, respectively.

Choices from the OLE have also been broadly distributed across degrees and across the different types of units on offer, including ethics and contemporary debates, global challenges, innovation and communication, and teamwork skills. Figure 2 shows the distribution of OLE unit enrolments as a function of the faculty of the degree in which students are enrolled and the faculty teaching their chosen OLE units. Choices demonstrate a high level of enrolment outside the faculty of degree, suggesting that students are indeed using the OLE to broaden their knowledge and skills.

Across all undergraduate students and degrees, the percentage of studies taken outside the faculty of a student’s degree has risen from 13.3% in 2017 to 16.8% in 2021.

Students have also relished the expansion of opportunities for experiential learning. As the data in Table 3 indicate, enrolments in industry and community project units (2018–2022) have risen sharply, increasing by 275% from 2018 to 2022.

**Table 3:** Undergraduate enrolments at the University of Sydney in industry/community project units, 2018–2022

Year	Enrolments
2018	4372
2019	5619
2020	8628
2021	10,076
2022	12,027

**Fig. 3:**
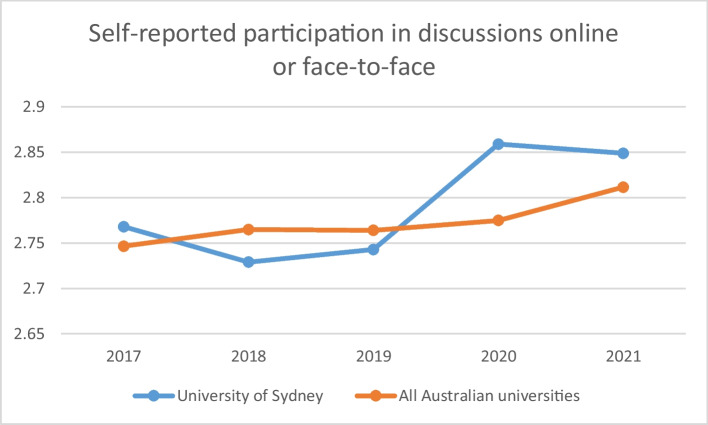
Mean undergraduate student responses on the Student Experience Survey item “Thinking about your < course > in < year > , how frequently have you participated in discussions online or face-to-face?”, by year. (response scale: 1 = never, 2 = sometimes, 3 = often, 4 = very often). Standard errors for means are less than .0125 (University of Sydney) and .0025 (all Australian universities)

Interactive classroom experiences are reflected in part by an item on the national Student Experience Survey, ‘I participated in discussions online or face-to-face’. Figure 3 presents the institutional data for the University of Sydney on this item from 2017 to 2021 together with the data aggregated across all universities. There is clearly a relative increase for the university in agreement to this item, especially from 2020, the first year of the pandemic, when a substantial professional development effort was mounted to support the commitment to interactive experiences during the move to remote learning.

A survey of student views on the purpose and importance of curriculum components undertaken in 2021 indicated that most students thought that most components were important. Importantly, however, the survey also made clear that more work was required to ensure that students had a broad and evidence-based understanding of the educational impact of each component. Student views of each component were, in fact, quite narrow (e.g. seeing improved employment prospects as the sole benefit of an industry and community project) and students were unaware of broader impacts on epistemological maturity, foundations for further learning, collaboration, and leadership. Students were also less positive about the OLE than other components, especially if they were completing two majors. The University is currently implementing a new advising program to support students to reflect on their aspirations and access evidence-based information on how to achieve them; these data will serve as important input. The findings also support the emphasis placed by Fischman and Gardner (2022) on ‘onboarding’ students to the educational purpose of their degree, ideally, with a series of regular check-ins to follow.

### Further assessment of the impact of educational change and the COVID-19 pandemic

In addition to understanding the impact of educational change at the University of Sydney in terms of students’ study patterns, it will be important in several years’ time to assess the impact on students’ perceptions of their educational experience and on graduate outcomes, including a range of indicators of graduate capability, such as capability assessments, graduate and employer perceptions of graduate capability, and labour market outcomes. It is arguably too soon for such assessment given the lag in availability of national survey data and the impact of the COVID-19 pandemic which arrived before any students undertaking the new curriculum at the University had graduated and affected both their educational experience and the labour market. Australia was no exception to the broader disruptive effects of the pandemic to higher education around the world, especially in 2020 and 2021. There was a sharp decline nationally in undergraduate student satisfaction with their overall educational experience in 2020, with the satisfaction level dropping from 78 to 80% in the years preceding 2020 to 69% in 2020 (QILT, 2021c). Only a partial recovery to 74% was recorded in 2021. Furthermore, the declines appeared to be greater in those parts of Australia that experienced longer periods of lockdown. Needless to say, understanding the impact of educational change in the presence of a variety of disruptive effects will be difficult, and a comprehensive view of the impact of educational change on students’ educational experiences and outcomes at the University of Sydney will likely require data from at least several more years and possibly longer, especially given that many international students are still experiencing travel restrictions in 2022 and are therefore continuing to study remotely.

## Discussion

In terms of implementation of educational change, the University community already recognises that some further change is likely to be required. For example, there is some way to go in embedding the development of *cultural competence* across the undergraduate curriculum, and a more systematic approach may be needed to ensure engagement with relevant ethical frameworks. There will be value in further expanding project, placement, and internship opportunities, and some change may be necessary to the enrichment program to ensure effective coordination with students’ other enrichment aspirations. The interdisciplinary opportunities in the third year continue to evolve, with more ‘curated’ interdisciplinary opportunities among proximal disciplines emerging alongside the Industry and Community Project Units described earlier.

In his recent book, Bok (2020) sets out some of the reasons why it is difficult to achieve the kind of change that the University of Sydney has sought to make. These reasons include the somewhat patchy nature of the relevant evidence base, the general reluctance of academic leaders to tackle undergraduate curriculum reform, and resistance among academic staff to make changes to the curriculum or to educational approaches. Like other universities who have refined the purpose of their undergraduate education, the University of Sydney community can take pride in a broadly successful program of change. However, Bok’s analysis raises the question of sustainability, especially for those components of the curriculum that require ongoing collaboration and co-ordination. Bok urges a resolve of purpose above all, but makes some practical suggestions as well. These include greater investment by government and universities in research in higher education; the infusion of academic communities with greater educational expertise; the inclusion of preparation for teaching as part of doctoral training programs; greater recognition and investment in professional staff and co-curricular programs that impact on student learning; and ongoing engagement of the academic community in questions of purpose and approach.

Bok’s (2020) focus on educational expertise and the need for more higher education research resonates strongly with the University of Sydney’s experience of change. Institutional educational change is only possible with the commitment and effort of expert educators distributed across the institution. Support for professional learning and an increase in educational expertise are therefore both vital.

In relation to research, we point to three ways in which a deeper research base would have been helpful even though the available evidence from the meta-analytic and synthetic studies cited earlier provided helpful guidance for the direction the University took. The first is a richer and more complete understanding of different forms of interdisciplinary experience and their impacts. The current evidence for impact is promising as we noted earlier, but arguably not yet as compelling as it needs to be to garner the breadth and strength of support to sustain it over long periods in our often devolved institutions (Davidson, 2017). The second is more ambitious research at the multi-institutional level that allows for an assessment of approach to change and impact across different student cohorts and in different organisational settings. This requires more co-ordinated, larger scale, multi-level, and multi-organisational higher education research programs, rather than post hoc aggregation of piecemeal research in single settings. It would clearly require up-front agreement on some key propositions worth exploring in this way as well as a high level of co-ordination and resource commitment, but for undertakings such as the one followed at the University of Sydney, would provide invaluable evidence. A third type of research supporting individual institutional change are detailed case studies that provide a richly contextualised picture of change, including longer term outcomes for students and reflections on successes and failures. We therefore hope that, once several additional years of data have accumulated, a detailed analysis of students’ educational experiences and outcomes can add to the picture of change at the University of Sydney.

## Data Availability

No new data were created for this study; rather, the aggregate data presented in the paper were drawn from internal institutional or national reports.
